# Case of Dislocation of the Processes of the Cervical Vertebræ

**Published:** 1857-01

**Authors:** Edwin R. Maxson

**Affiliations:** Geneva, N. Y.


ART. V.—*Case of Dislocation of the Processes of the Cervical Vertebræ.* By EDWIN R. MAXSON, M. D., of Geneva, N. Y.

I was called during the night of the 28th of October last, to see a child about nine years old, the daughter of Mr. S., of this village, said to be suffering from convulsions, and some difficulty about the neck.

I found the child in bed with the face turned toward the left shoulder, and without the ability to move or turn the head in any other direction.

The eyes were apparently fixed, and the countenance presented a very unnatural appearance, the face being of a pale lead color, with a much darker appearance below the eyes.

The history of the case, as stated to me by the parents, was as follows:

On the morning of the 27th, which was about forty hours before I was called, the girl was playing with a smaller child, when, by a sudden and probably a forcible turn of the face toward the left shoulder, she suffered from a pain in the back of the neck, and also became faint, and found herself unable to turn the head back to its natural position.

The mother being immediately called, placed her in the most comfortable position she could, and, as the faintness grew less, she hoped that the difficulty might subside, and the head come right, without any medical or surgical aid.

She continued much the same during that day, but at night, when she was moved to her bed, she became faint, restless, and irritable, and had but little rest during the night. The second day she continued much as on the first, only that her countenance grew worse, and she complained more of her neck.

Late in the evening of the second day, in attempting to move her again to her bed, they, by accident, turned the face a little more toward the left shoulder, as they supposed, when she was severely convulsed for a considerable time: after which she became very faint, and could not bear to be raised up much in bed.

It was at this time that I was called to see her, as I have before stated.

The face had not been turned from toward the left shoulder since the accident occurred, and though the convulsions had subsided when I arrived, there was more or less spasm of the muscles, especially on any attempt to raise her up, or to change her position.

From a history of the case, together with her symptoms at the time, I suspected a dislocation of the oblique processes of some one of the cervical vertebræ, as described by Samuel Cooper, and others. (See S. Cooper's Dictionary of Practical Surgery, by Reese, article Dislocation, page 308.)

In order to form, if possible, a correct diagnosis in the case, I passed my hand along the spinous processes of the cervical vertebræ, which, the child being spare, I could distinctly feel, till I reached the fifth or sixth, where I discovered an irregularity: the spinous process of the fifth or sixth cervical vertebræ appearing to the right of the one below.

From all the symptoms I now felt confident that there was displacement of the processes of the fifth or sixth cervical vertebræ, to such an extent as to slip the transverse or oblique processes, or both, of one vertebra, from the corresponding processes of the vertebra below; and, that the transverse or oblique processes, or both, of one vertebra, were caught against the corresponding processes below: and hence the inability to turn the face from toward the left shoulder.

I stated to the parents what I believed to be the real condition, and also the danger of convulsions, or immediate death, should an attempt be made to replace it; and still further, the great danger of delay in the case; after which they expressed a wish that I would do what I could for her, as they feared she could not endure the worriment of further counsel in her case that night.

I accordingly resolved to make an attempt at reduction at once; and determined, should I fail, to take the advice of my friend, Dr. H. A. Potter, an eminent surgeon of this village, as soon as she could become quiet, and her condition would permit of it.

I grasped the head, with both hands, and proceeded according to Desault's method, only I first carried or turned the face very gently a little further toward the left shoulder, to, if possible, disengage the process; then lifting or extending the head, I turned the face very gently toward the right shoulder, when the difficulty was at once overcome, and she exclaimed, "I can move my eyes."

Her countenance soon acquired a more natural appearance; the faintness passed off; she rested quietly through the night; had no return of the difficulty, and needed only an emollient anodyne to soothe the irritation and slight swelling which remained at the point of injury.

GENEVA, N. Y., Nov. 10th, 1856.

